# Implications of Possible HBV-Driven Regulation of Gene Expression in Stem Cell-like Subpopulation of Huh-7 Hepatocellular Carcinoma Cell Line

**DOI:** 10.3390/jpm12122065

**Published:** 2022-12-14

**Authors:** Ayse Banu Demir, Domenico Benvenuto, Bilge Karacicek, Yasemin Erac, Silvia Spoto, Silvia Angeletti, Massimo Ciccozzi, Metiner Tosun

**Affiliations:** 1 Department of Medical Biology, Faculty of Medicine, Izmir University of Economics, 35330 Izmir, Turkey; 2 Faculty of Medicine, University Campus Bio-Medico of Rome (UCBM), 200 Rome, Italy; 3 Izmir Biomedicine and Genome Center (IBG), Dokuz Eylul University Health Campus, 35340 Izmir, Turkey; 4 Department of Pharmacology, Faculty of Pharmacy, Ege University, 35100 Izmir, Turkey; 5 Diagnostic and Therapeutic Medicine Division, Fondazione Policlinico Universitario Campus Bio-Medico, 200 Rome, Italy; 6 Clinical Laboratory Science Unit, Faculty of Medicine, University Campus Bio-Medico of Rome (UCBM), 200 Rome, Italy; 7 Clinical Laboratory Research Unit, Fondazione Policlinico Universitario Campus Bio-Medico Via Alvaro del Portillo, 200 Rome, Italy; 8 Medical Statistics and Molecular Epidemiology Unit, Faculty of Medicine, University Campus Bio-Medico of Rome (UCBM), 200 Rome, Italy; 9 Department of Medical Pharmacology, Faculty of Medicine, Izmir University of Economics, 35330 Izmir, Turkey

**Keywords:** hepatocellular carcinoma, HBx, miR3653, epithelial–mesenchymal transition (EMT), molecular evolution

## Abstract

Elevated levels of STIM1, an endoplasmic reticulum Ca^2+^ sensor/buffering protein, appear to be correlated with poor cancer prognosis in which microRNAs are also known to play critical roles. The purpose of this study is to investigate possible HBV origins of specific microRNAs we identified in a stem cell-like subpopulation of Huh-7 hepatocellular carcinoma (HCC) cell lines with enhanced *STIM1* and/or *Orai1* expression that mimicked poor cancer prognosis. Computational strategies including phylogenetic analyses were performed on miRNome data we obtained from an EpCAM- and CD133-expressing Huh-7 HCC stem cell-like subpopulation with enhanced *STIM1* and/or *Orai1* expression originally cultured in the present work. Results revealed two putative regions in the HBV genome based on the apparent clustering pattern of stem loop sequences of microRNAs, including miR3653. Reciprocal analysis of these regions identified critical human genes, of which their transcripts are among the predicted targets of miR3653, which was increased significantly by *STIM1 or Orai1* enhancement. Briefly, this study provides phylogenetic evidence for a possible HBV-driven epigenetic remodeling that alters the expression pattern of Ca^2+^ homeostasis-associated genes in *STIM1*- or *Orai1* overexpressing liver cancer stem-like cells for a possible mutual survival outcome. A novel region on HBV-X protein may affect liver carcinogenesis in a genotype-dependent manner. Therefore, detection of the viral genotype would have a clinical impact on prognosis of HBV-induced liver cancers.

## 1. Introduction

Hepatitis B virus (HBV), a hepatotropic DNA virus with different genotypes, replicates via reverse transcriptase and incorporates into the host genome at early steps of clonal tumor expansion, which eventually leads to hepatocellular carcinoma (HCC) [1,2,3]. In Huh-7 HCC cell lines, epithelial cell adhesion molecule (EpCAM)- and Prominin-1 (CD133)-co-expressing cell subpopulations were shown to possess stem cell-like properties, as they frequently caused tumor development in immunocompromised NOD/SCID mice xenograft models [4,5,6]. For simplicity, these tumor-initiating cell subpopulations, therefore, are termed cancer stem-like cells (CSCs) in the present study. Enhancement of endoplasmic reticulum (ER) transmembrane stromal interaction molecule 1 (STIM1) and/or Calcium Release-Activated Calcium Modulator 1 (Orai1) expression in Huh-7 CSCs increased store-operated Ca^2+^ entry (SOCE) and proliferation rate along with upregulated MDR1 expression, each associated with poor cancer prognosis [7]. SOCE-mediated increase in cytosolic Ca^2+^ concentration appears to be among the initial events in HBV-associated hepatocarcinogenesis [8,9].

The presence of HBxAg, an *HBV-*X** gene-encoded protein with a wide spectrum of functions, including viral life-cycle, is required for effective infection and oncogenic potential [1]. HBx reportedly increases intracellular Ca^2+^ levels via SOCE, stimulates cell proliferation and induces HBV replication in HCC [8,10]. Therefore, HBx could play essential roles in HBV-associated HCC by interacting with the STIM1 and Orai1 complexes to maintain SOCE, as well as targeting key microRNAs (miRNAs) in host cells [9,11,12,13,14]. In viral infections, miRNAs can alter viral replication processes by regulating the viral gene expression and cellular factors associated with HBV pathogenesis [15]. Many host miRNAs appear to be associated with HBV-induced genomic instability, and their expression profile is altered in stable HBV expression [16].

A possible causative role of STIM1 elevation in different types of cancer makes it an attractive chemotherapeutic target [9,17]. This study investigates a possible phylogenetic relationship between HBV and certain miRNAs, whose expression patterns were altered by STIM1 and/or Orai1 enhancement in Huh-7 HCC CSCs. In order to define key regulatory miRNAs, we upregulated the poor prognosis markers, STIM1 and/or Orai1, in EpCAM- and CD133-expressing Huh-7 cells, as these HCCs have been shown to be highly tumorigenic in mouse xenograph models [4,5,6]. Finally, in order to evaluate their possible viral origin, we performed in silico experiments, including phylogenetic analysis, on significantly altered miRNAs whose expression levels were significantly altered by STIM1 and/or Orai1 enhancement in Huh-7 HCC CSCs (Table S1, Figure S1). Our analyses revealed certain viral genomic segments as possible origins of some “human” miRNAs, which are critical for cancer cell survival and possibly edited through early evolutionary events in order to modulate the host’s epigenetic defense system on behalf of the virus in a genotype dependent manner.

## 2. Materials and Methods

### 2.1. Cell Culture

Huh-7 HCC cell lines, originally from Dr. Jack Wands Laboratory (Massachusetts General Hospital, Boston, MA, USA), were tested for authenticity via DNA profiling (Applied Biosystem’s Identifier kit, PN 4322288) at DNA Sequencing & Analysis Shared Resource, University of Colorado Cancer Center. The cells’ identity was reconfirmed by Idexx Bioresearch Company (Kornwestheim, Germany) before conducting present study. Mycoplasma contamination was monitored regularly in our laboratory using MycoAlert Mycoplasma Detection Kit (Lonza, Basel, Switzerland). All cell culture processes were performed as described earlier [7].

### 2.2. Separation of Huh-7 CSCs

Cells were prepared for a fluorescence-activated cell sorter (FACS Aria III, BD Biosciences) [7]. A separation process was carried out based on differential fluorescence emission properties of labeled antibodies targeting EpCAM and CD133 (EpCAM-FITC, CD133-PE, Miltenyi). EpCAM- and/or CD133-expressing cells were collected separately in FBS-containing tubes. The percentages of EpCAM(+)/CD133(+) cell populations were recalculated by flow cytometry (FACSCalibur, BD Biosciences, Franklin Lakes, NJ, USA) on the same day. Experiments were performed in triplicate.

### 2.3. Plasmid Transfection

Cells incubated for 24 h with X-tremeGENE HP DNA Transfection Reagent (Roche, Basel, Switzerland) on 6-well cell culture plates (105 cells/well) were transfected with plasmid DNA (MO70-STIM1-eYFP, pDEST501-Orai1-CFP and corresponding empty vector backbone as control) for an additional 1 h [7]. Plasmids were kindly provided by Dr. M. Trebak (Penn State University). Experiments were performed in triplicate.

### 2.4. Invasion Assay

Forty-eight hours after transfection, control and STIM1-OE and Orai1-OE Huh-7 HCC CSCs were cultivated in 6-well plates (Corning, 8 µm-pore diameter, 6 × 10^4^ cell/well) to investigate invasion and migration characteristics. After 24 h, cells were stained with RAL Diff-Quik staining kit (Siemens, München, Germany) according to the manufacturer’s instructions, and migrating cells were counted under an inverted microscope (Olympus IX71, Tokyo, Japan). Experiments were performed in triplicate.

### 2.5. PCR Analyses for EMT Markers 

mRNA expression levels of target genes (E-cadherin, N-cadherin and Vimentin) were determined using qRT-PCR (LightCycler 1.5, Roche). cDNAs were synthesized (Transcriptor High Fidelity cDNA synthesis kit) using RNA samples (1 µg) isolated from the cells (HighPure RNA Isolation Kit, Roche) under isolation cabinet with laminar air flow, and confirmed for their lack of genomic DNA contamination by omitting reverse transcriptase in an additional sample during the reverse transcription procedure. The High Pure RNA Isolation kit used to isolate total RNA also involved an integrated DNase digestion step to avoid any residual genomic DNA. FastStart DNA Master SYBR Green I Kit (Roche) was used to determine mRNA expression levels (Table S4). Serial dilutions of standard 18S rRNA cDNA–containing plasmid with known copy numbers were used in each PCR run to construct linear regression curves to calculate mRNA concentrations specifically for each study, as described earlier [7]. Experiments were performed in triplicate.

### 2.6. miRNome Analysis

Total RNA was extracted from cell samples using Total RNA Purification Kit (Norgen) which also allows the collection of small non-coding RNAs, including miRNAs, according to the manufacturer’s instructions. RNA concentrations in each sample were determined by Nanodrop (Thermo Fischer Scientific, Waltham, MA, USA). Isolated total RNA samples were analyzed (Agilent Bioanalyzer 2100, Santa Clara, CA, USA) to calculate the “RNA Integrity Number” (RIN) (1 to 10), of which the value close to 10 showed the robustness of the RNA content. Therefore, the samples with RIN ≥7 were used in library preparation. A stepwise procedure was performed to prepare cDNA libraries from small RNA samples via ligation of 3′ and 5′ adapters, excision of the corresponding constructs from the gel, purification, and cDNA preparation via RT reaction, according to the manufacturer’s instructions. Regarding the critical step in gel electrophoresis, the portion between the lower limit of the 160 bp band and the upper limit of the 145 bp band was recovered from the gel to collect the cDNAs of interest, as the size of the original mature miRNAs (app. 22 nt) increased to 147–150 nt with the addition of 3′ and 5′ adapters.

Next-generation sequencing (Illumina NextSeq 500, San Diego, CA, USA) was performed (DONE Genetics & Bioinformatics, Istanbul, Turkey) on cDNA libraries prepared from total small RNA samples isolated in our laboratory. In the Solexa sequencing method used by Illumina, DNA fragments were first placed on flow cells and amplified locally to give a high accuracy signal at the site of settlement (bridge amplification). As a result, millions of clusters were formed on the flow cell. Fragment clusters formed on the flow cell were sequenced to read one base per cycle. In each cycle, DNA fragments were labeled with 4 different dyes and exposed to 4 different dinucleotides by the sequencing-by-synthesis method developed by Illumina. In each cycle, the clusters were visualized by fluorescence camera following binding of the appropriate dinucleotide. Images from each cluster were combined, and the nucleotide sequences were deduced from the emission characteristics of the respective clusters. 

Quality control: the efficiency of the sequencing process was assessed by using the FASTQC program (www.bioinformatics.babraham.ac.uk). Reading filters and trimming: During the sequencing process, poor quality base readings in the FastQ readout data were excluded from readings to avoid false positive results in subsequent analysis steps. Trimmomatic application (http://www.usadellab.org/cms/?Page=trimmomatic, accessed on 21 October 2015) was used for quality filters and trimming procedures. In order to detect miRNA sequences, the identified individual sequences were matched with BLAST via miRBase (www.mirbase.org, accessed on 23 October 2015). If the examined sequences matched the miRNA sequences exactly in the miRBase, the corresponding sequence was considered as a miRNA candidate. The sequencing experiments were performed in triplicate.

### 2.7. RNA-Seq Data Analysis 

NextSeq 500 (Illumina) sequencing data were generated in *.bcl format. These files contained the location and IDs of each DNA cluster on the flow cell, and the emission characteristics. From these data, *.fastq files were obtained by using Bcl2fastq v.2.1 (Illumina) program. At the same time, fastq data were divided into different samples according to the index data (Demultiplexing).

### 2.8. Phylogenetic Analysis of miRNAs

Stem loop regions of the miRNAs, whose expression levels were significantly altered in CSCs due to STIM1 and/or Orai1 overexpression compared to their corresponding control Huh-7 cells, were retrieved from MirBase Database (http://www.mirbase.org/search.shtml, accessed on 1 September 2019). A dataset containing the stem loop sequences of 73 miRNAs was built. In order to compare these sequences with the HBV genome, the dataset was constructed including these miRNAs and the HBV genotypes A, B, C, D, E, F, G and H. The sequences were aligned using MAFFT server (https://mafft.cbrc.jp/alignment/server/, accessed on 2 September 2019) and then manually edited using Aliview software. Then, they were divided into sub datasets containing different DNA regions with the highest degree of sequence alignment, and tentatively named “Region 1” and “Region 2” for HBV. A neighbor-joining tree (NJ), a minimum parsimony tree (MP), a Bayesian phylogenetic tree (BP), minimum evolution Tree (ME) and a minimum spanning tree (MST) plot were drawn for each region. MEGA-7 software was used to draw ME, NJ and MP trees using 1000 bootstrap replications, keeping the other parameters as default, Bayes using 1 million Markov chain Monte Carlo generations was used for the BP and an in-house script in R based on the Kruskal genetic distance matrix was used for MST. Pairwise *genetic distances* were calculated by *MEGA* software (version *7.0*). The trees were compared and discussed based on the miRseq data obtained from Huh-7 HCC stem cells. Unresolved trees were discarded, and miRNAs that changed upon plasmid-only transfection were excluded from further analysis.

The HBx regions aligned with some parts of the stem-loop sequences were analyzed separately due to the presence of numerous nucleotide mismatches. The resolved trees (MST and ME) were performed independently for distinct regions, and only miRNAs clustered close to the viral genome in both approaches were evaluated. Due to sequence shortness, clusters determined by two different mathematical models were used to confirm the similarity between the human miRNAs and the putative regions of the HBV genome. All the phylogenetic analyses were performed in triplicate for confirmation, and similar clusters were obtained each time.

### 2.9. Genomic Database Analysis

HBV Regions 1 and 2 were compared with the HBV genome (https://hbvdb.ibcp.fr/HBVdb/HBVdbGenome, accessed on 1 September 2019) in order to identify with which genetic region in the HBV genome they overlapped, and a further BLAT alignment algorithm was used to identify a possible region within the human genome (hCG38) in which these regions may be aligned (http://genome.ucsc.edu/cgi-bin/hgBlat, accessed on 5 September 2019). BLAT analysis was performed instead of BLAST, since the sequences were too short for BLAST analysis. 

### 2.10. Putative Protein Structure Homology Analysis

Based on the HBx protein file (https://swissmodel.expasy.org/repository/uniprot/Q156X2, accessed on 10 October 2019), the putative “Region 1” (amino acids between 10 and 34) and “Region 2” (amino acids between 117 and 138) on HBx protein were labeled using the RasMol program for Windows (RasWin). In order to find protein structures similar to the identified HBV regions, homologous structural templates were searched and validated using the template search tools HHpred [18] and SwissModel [19]. Three-dimensional structures were constructed using PyMOL (The PyMOL Molecular Graphics System, Version 2.0 Schroedinger, LLC). The QMEAN Z Score and GMQE (Global Model Quality Estimation) were used to calculate the degree of nativeness of the structural feature observed in the model on a global scale and to estimate the quality of the 3D model, respectively [20].

### 2.11. Cancer Cell Line Database Analysis

The expression patterns of miR3653 and its target genes were evaluated by using the Liver Cancer Cell Line Database [21], the Oncomine Website (for Barretine Cell Line Dataset, Gyorffy Cell Line Dataset, Wooster Cell Line Dataset) and the NCBI Gene Expression Omnibus (GEO) Database (for the datasets GSE112788, GSE79232, GSE97172, GSE85274, GSE88812, GSE132119, GSE73219 and GSE36139). The miR3653 expression patterns among different cancer cell lines were also analyzed using the Harmonizome database.

### 2.12. Statistics

miRNome analyses: One-way ANOVA and post-hoc Tukey tests were performed to compare two groups. Probes that provided a value of *p* < 0.05 between groups were used for further evaluation. FC (fold change) analysis was performed, and changes greater than 1.5-fold were specified as increases (plus values) and decreases (negative values) for each probe, as shown in the Supplementary Tables.

PCR and cell migration assay: Data are expressed as mean ± standard error of the mean. ‘‘n’’ represents the number of samples. Statistical significance between the means of the two groups was evaluated using Student’s *t*-test (unpaired data) using Graph Pad Prism software (GraphPad, San Diego CA, USA) and *p* < 0.05 considered statistically significant (Figure S2).

## 3. Results

### 3.1. miRNA Profile upon Orai1 and STIM1 Overexpression

**Figure 1:**
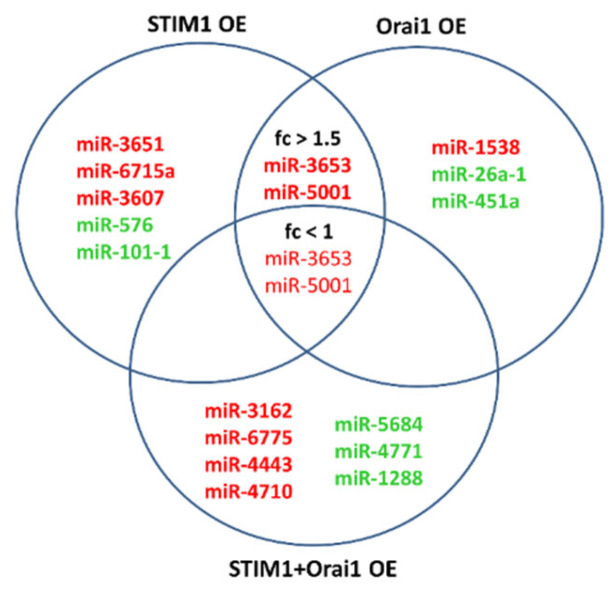
Venn diagram showing candidate miRNAs with significantly altered expression levels in STIM1 and/or Orai1-overexpressing Huh-7 HCC stem-like cells. miRSeq data (Illumina NextSeq 500) were obtained from Huh-7 cancer stem-like cell samples treated with overexpression (OE) constructs for STIM1, Orai1 and both (STIM1&Orai1). Upregulated and downregulated miRNAs were evaluated compared to each condition’s appropriate plasmid-only controls and are shown in red and green letters, respectively. Micro RNA sequencing was performed by Illumina NextSeq 500 NGS platform. Experiments were performed in triplicate. fc: fold change.

Based on our miRNome data, enhancement of STIM1 and/or Orai1, which mimicked poor HCC prognosis, significantly altered the expression levels of seventy-three miRNAs in Huh-7 CSCs, of which thirty-three were substantially changed comparable to the corresponding controls or non-CSCs (Table S1). Among these, miR3653 and miR5001 were the only miRNAs that were significantly upregulated (Fold Change [FC] > 1.5, in red) in all HCC CSC samples (Figure 1).

### 3.2. Phylogenetic Analysis of miRNAs 

**Figure 2:**
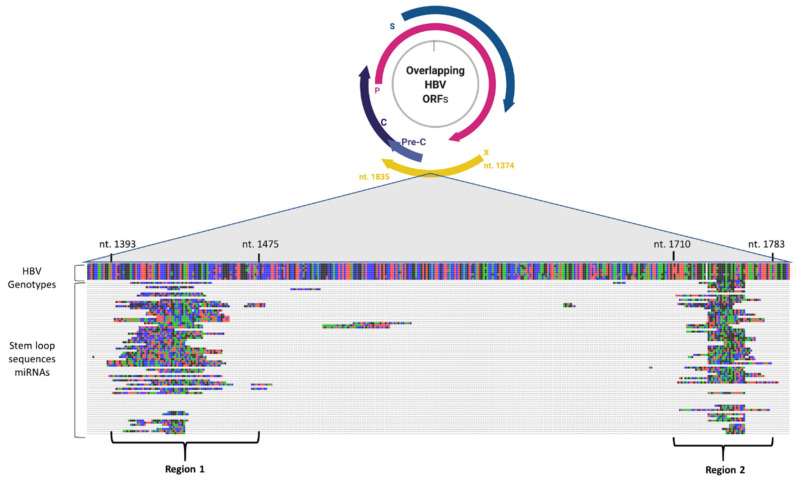
Representation of HBV genome along with overlapping HBV Open Reading Frames (ORFs). The locations of “Region 1” (from HBV nucleotide 1393 to 1475) and “Region 2” (from HBV nucleotide 1710 to 1783) are shown on the HBx coding region (from HBV nucleotide 1374 to 1835), where the stem-loop sequences of many miRNAs are partially aligned through multiple sequence alignment. Only the stem-loop sequences of miRNAs were aligned to match HBV genome, as no direct alignment patterns were found for their corresponding mature miRNAs. (The schematic representation was created by BioRender.com).

Stem-loop sequences of the miRNAs whose expression levels have changed significantly due to *STIM1* and/or *Orai1* enhancement, were used in phylogenetic analyses to investigate their viral origins. Multiple alignment analyses revealed two putative HBV segments, “Region 1” and “Region 2” (Figure 2, Table S2), in which most of the stem-loop sequences were aligned. 

Due to the presence of many unmatched nucleotides between these two regions, further phylogenetic analyses were performed separately for each region. Among all phylogenetic analyses, only the minimum evolution (ME) and minimum spanning tree (MST) algorithms were able to draw resolved trees, whereas the neighbor-joining (NJ), maximum parsimony (MP) and Bayesian parsimony (BP) algorithms were not operational. Therefore, only the stem loop sequences of miRNAs clustered close to HBV genotypes in both trees were used for further analyses.

**Figure 3:**
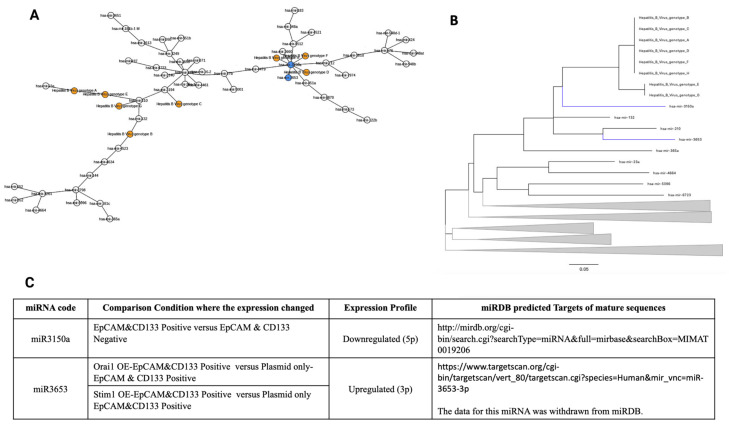
Phylogenetic analysis of HBV Region 1 for miRNAs expressed differentially in Huh-7 HCC CSCs. (**A**) The minimum spanning tree (MST) and (**B**) the minimum evolution Tree (MET) for the miRNAs and Region 1 of different HBV genotypes. In MST, the HBV genotypes (orange) and the miRNAs found close to HBV Region 1 both in MST and MET (blue) are shown. (**C**) Expression profiles and target details of the miRNAs found close to HBV region 1 in MST and MET. None of the miRNAs listed were changed upon plasmid-only transfection. HCC CSCs: hepatocellular carcinoma stem-like cells; OE: overexpressed; EpCAM(+)/CD133(+); miRDB: an online database for miRNA target prediction and functional annotations.

miR3150a clustered close to the HBV “Region 1” in both trees (Figure 3A,B). miR3150a(5p) was downregulated in CSCs (EpCAM- and CD133-expressing Huh-7 cells) compared to that of parental HCC cells.

**Figure 4:**
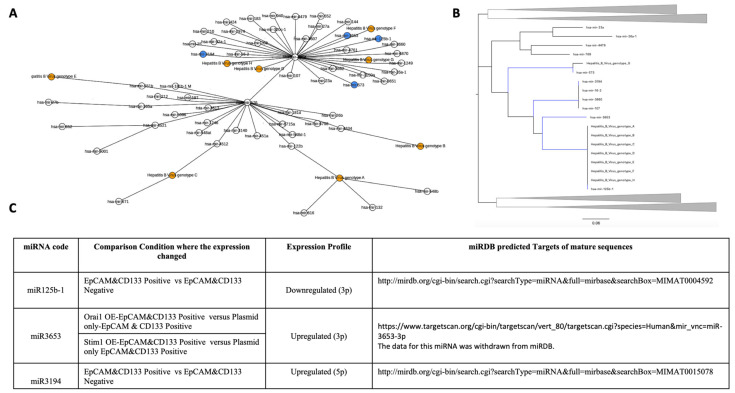
Phylogenetic analysis of HBV Region 2 for miRNAs expressed differentially in Huh-7 HCC CSCs. (**A**) The minimum spanning tree (MST) and (**B**) the minimum evolution tree (MET) for the miRNAs and Region 2 of different HBV genotypes. In MST: the HBV genotypes (orange) and the miRNAs found close to HBV Region 2 both in MST and MET (blue) are shown. (**C**) The expression profiles and the target details of the miRNAs found close to HBV region 2 in MST and MET. HCC CSCs: hepatocellular carcinoma stem-like cells; OE: overexpressed, EpCAM(+)/CD133(+); NSCSs: non-stem cancer cells, EpCAM(-)/CD133(-).

Regarding the miRNAs clustered within HBV “Region 2” (Figure 4); miR125b-1(3p) was downregulated in CSCs compared to parental Huh7 HCC cells, while miR3194(5p) was upregulated. 

miR3653(3p) clustered both in “Region 1” and “Region 2” and was upregulated in both *STIM1*- or *Orai1*-overexpressing CSCs compared to that of the control CSCs (Figure 3C and Figure 4C).

### 3.3. Genomic Database Analysis of Putative HBV Regions

The identified HBV regions were used in BLAT analysis against the human genome (hCG38) (http://genome.ucsc.edu/cgi-bin/hgBlat, accessed on 5 September 2019) in order to evaluate whether these specific viral regions were also present in the human genome. Although HBV “Region 1” did not match the human genome, “Region 2” showed several significantly matching regions in a genotype-dependent manner (Table S3). 

### 3.4. Structural Homology Analysis

**Figure 5:**
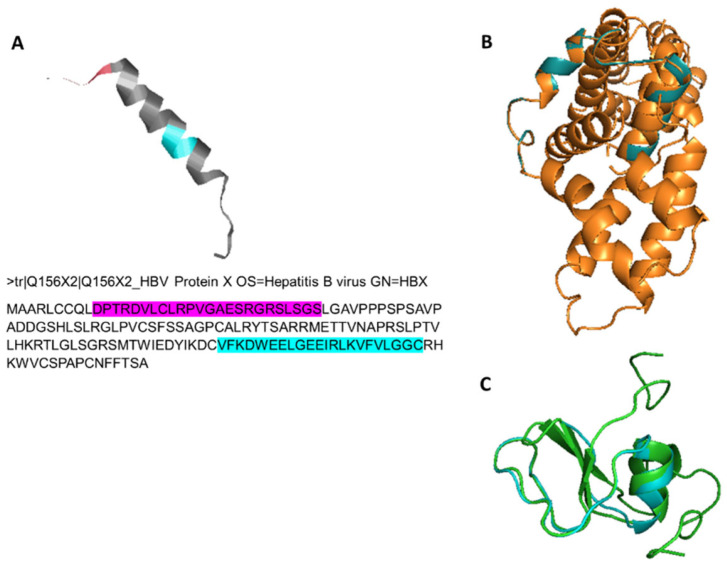
Ribbon diagram for putative (**A**) Region 1 (pink) (from HBV amino acid 469 to 490; HBx amino acid 10–34) and Region 2 (cyan) (from HBV amino acid 574 to 590; HBx amino acid 117–138) on HBx protein. Superimposed homologies for (**B**) 3lra.1.A structure, membrane-associated guanylate kinases p55 subfamily member 7 (Mpp7, orange), where this heterodimer contains 13 α-helix and Region 2 for HBV genotypes A, B, E, G and H (green), and (**C**) 1ksq.1.A structure, Latent Transforming Growth Factor β-Binding Protein 1 (TGFβ-LTBP-1, green), showing a monomeric core structure with two α-helices covered by three β-sheets, and Region 2 of HBV genotypes C, D and F (cyan).

Locations of “Region 1” and “Region 2” on HBx protein were shown in Figure 5A. For homology analysis, these regions within all different HBV genotypes were used as separate reference sequences to investigate whether any homology existed with human proteins. Homologies were primarily identified for genotype F (DQ823095). The calculated QMEAN z Score was statistically significant (−4 < QMEAN z Score < 0), and the GMQE (Global Model Quality Estimation) score used to estimate the quality of the three-dimensional model was 0.4, considered an average value for heterodimeric models constructed with short sequences according to the software algorithm guidelines. These results showed the convenience of the model for our purpose. Among all HBV genotypes tested, homology models were found only for “Region 2” templates. These models yielded two candidate proteins; membrane-associated guanylate kinases p55 subfamily member 7 (MPP7) (PDB code 3lra.1.A) for HBV genotypes A, B, E, G and H (Figure 5B), and Latent Transforming Growth Factor β Binding Protein 1 (TGF β-LTBP-1) (PDB code 1ksq.1.A) for HBV genotypes C, D and F (Figure 5C).

The list of GEO datasets of HCC cell lines was also analyzed for Mpp7 and β-LTBP expressions at the RNA level (Supplementary Table S5), both showing up to a 1.5- to 2-fold change in differential expression levels in other HCC cell lines. The apparent negative correlation between Mpp7 and β-LTBP expressions in SNU449 and Hep3B cells (poorly and well-differentiated HCC cell lines, respectively) deserves further investigations (GSE36139 “GPL15308 Platform”; GSE85274 “GPL13667 Platform”). A differential expression pattern of these genes in different HBV infections would identify if HBV genotypes play role in this process.

### 3.5. E-Cadherin Expression Changes

After finding high homology among Region 2 and certain parts of MPP7 and TGF β-LTBP, which are known to play a role in EMT, we tested the effects of *STIM*1 and/or *Orai1* enhancement in Huh-7 HCC CSCs on “Epithelial to Mesenchymal Transition (EMT)” and “Mesenchymal to Epithelial Transition (MET)”. The transcription levels of the epithelial (*E-cadherin*) and mesenchymal (*N-cadherin* and *vimentin*) markers were determined. *E-cadherin* expression was significantly increased both by *STIM1* and *STIM1* plus *Orai1* enhancement (Figure S2), while no significant change was observed in *N-cadherin* and *vimentin* (not shown).

### 3.6. Expression Patterns of miR3653 Target Genes 

In order to identify the expression patterns of the miR3653 target genes, the Liver Cancer Cell Line Database was analyzed [21]. The analysis showed no difference in the expression pattern of target genes in liver cancer samples with unaltered miR3653 expression levels. Only the *CAPN13, CES4A, B4GALNT3* and *MUC20* expressions were high in Huh6 compared to other cell lines, and *B4GALNT3* expression was higher in Hep3B, HepG2, SNU182 and SNU475 cells (Figure S3). This is a significant observation, as we investigated miR3653 expression in Huh-7 HCC CSC cells by enhancing *STIM1* and *Orai1* expression, which mimics poor cancer prognosis, instead of using parental EpCAM(-)/CD133(-) Huh-7 cancer cells. Therefore, miR3653 may only be expressed in cell subpopulations transformed into CSCs. 

We also analyzed several cell line datasets in Oncomine and NCBI GEO databases in order to see the expression patterns of miR3653 targets in different cancer cell lines besides the different HCC cell lines. According to these data, the expression patterns of these genes either increased or decreased in different cancer cell lines, and some of the genes, including MUC20 and B4GALNT3, showed decreased expression in HCC cell lines compared to other cancer cell lines. 

The Harmonizome Database showed different miR3653 expression levels among different cancer cell lines, with the highest levels in HCC cells, such as Huh7, SNU449 and JHH7 cells [22,23]. 

## 4. Discussion

Multiple alignment results of the stem loop nucleotide sequences of miRNAs revealed two putative HBV segments, which we proposed as “Region 1” and “Region 2”, where most of these sequences were partially clustered in the HBx-region of HBV genome (https://hbvdb.ibcp.fr/HBVdb/HBVdbGenome, accessed on 1 September 2019. Previously, miR3653 expression levels were shown to be significantly lower in HCC cells compared to those of non-cancerous hepatocytes, and its overexpression was shown to inhibit the growth and metastatic ability of HCC cells [24]. This appeared to mimic the dormant state of CSCs. In our study, besides being significantly upregulated by enhancement of *STIM1* and/or *Orai1* in CSCs, miR3653 was the only miRNA whose stem-loop sequence clustered within both “Region 1” and “Region 2”. In addition, its predicted targets have essential roles in carcinogenesis (https://www.targetscan.org/cgi-bin/targetscan/vert_80/targetscan.cgi?species=Human&mir_vnc=miR-3653-3p, accessed on 11 December 2022). 

BLAT analysis of the identified HBV sites against the human reference genome showed that an approximately 20–22 nt segment of viral Region 2 was found within the intronic regions of some target genes (Table S3). Evaluation of 192 DNA sequences from tumor samples and corresponding non-tumor samples revealed the direct repeat region (DR1), the most common fusion breakpoint of the HBV genome [25]. Therefore, we propose that integration of only the HBV “Region 2” into the human genome, unlike “Region 1”, may result from its close proximity to DR1. Potential cellular roles of six significant estimated miR3653 targets (CAPNs, CES4A, CEP76, MUC20, B4GALNT3 and MCU) found to be within the Region 2 integration region were discussed below in terms of HBV virulence and carcinogenesis. 

Calpains (*CAPN*s) are a family of Ca^2+^-activated nonlysosomal intracellular cysteine proteases that cleave STIM1 [26]. STIM1 and SOCE are linked to angiogenesis and cancer cell survival, both indicating tumor aggressiveness and poor prognosis [27]. Suppression of calpain by upregulated miR3653 in STIM1-enhanced HCC CSCs may have an additional survival outcome for the host. Certain parts of “Region 2” of HBV genotype A and F were found within the intronic region of Carboxylesterase 4A (*CES4A)*. Carboxylases encoded by this gene are responsible for the transesterification of certain endogeneous substrates and detoxification of various xenobiotics and drugs (https://www.genecards.org/cgi-bin/carddisp.pl?gene=CES4A, accessed on 6 July 2020). This gene was shown to be hypermethylated in HCC tissues [28]. Upregulation of miR3653(3p) in STIM1- and/or Orai1-overexpressing CSCs may suggest an additional epigenetic regulation of this enzyme in HCC. Hypermethylation of this gene and its posttranscriptional suppression by miR3653 can make cells vulnerable to xenobiotics. This dual epigenetic control could be a partial removal of the host defense system on behalf of the invading organism. Centrosomal protein 76 kDa *(CEP76)* appears to suppress centriole amplification, suggesting that elevated miR3653 may contribute to the dormancy of liver CSCs via *CEP76*, as centriole numbers are strictly controlled, and an increase in their copy number is considered among the hallmarks of cancer [29]. Overexpression of Mucin 20 cell surface associated (*MUC20*), which is considered a prognostic marker in renal cancers (https://www.proteinatlas.org/ENSG00000176945-MUC20, accessed on 6 July 2020), is also involved in the uncontrolled metastasis of colorectal cancer [30]. Some mucins have also been shown to be operational in primary liver lesions and HCC [31]. Integration of a certain segment of HBV genotype C in intronic regions of *MUC20* and upregulation of miR3653 targeting *MUC20* may make HBV-induced carcinogenesis liver resident, which is quite consistent with the hepatotropic property of the virus. This may explain why HCC does not metastasize extrahepatic tissues until advanced intrahepatic tumor stage IVA [32]. Certain parts of “Region 2” of HBV genotype D were found within the intronic region of Beta-1,4-N-acetyl-galactosaminyl transferase 3 (*B4GALNT3),* which was shown to be overexpressed in colon cancer cells regulating the stemness [33]. Knock-down of *B4GALNT3* led to suppression of the malignant phenotype [33]. Upregulation of miR3653(3p) in STIM1- and/or Orai1-overexpressing CSCs may, therefore, be a host defense response against the malignancy. This integration appears to be a part of the evolutionarily triggered survival strategy of human cells hosting HBV. Mitochondrial Ca^2+^ uniporter (*MCU*), a transmembrane protein that allows Ca^2+^ uptake into mitochondria, and its regulatory components *MICU1* and *MICU2* are among the targets of miR3653. Upregulation of miR3653 by *STIM1* enhancement may account for the survival of the CSCs by precluding Ca^2+^-induced apoptosis carried out by mitochondria. On one hand, overexpression of STIM1 increases the Ca^2+^ storage capacity of ER; on the other hand, miR3653 guarantees CSC survival. Consistently with the other miR3653 targets discussed above, downregulation of MCU also appears to be a part of the survival strategy of the host cells.

Within the in silico analysis of the present study, the putative “Region 2” was also found in intronic regions of *CASC8*, *SLC32A1* and *RPL23AP5*, which were previously shown to harbor integrated HBV genome sections in HCC patients [32]. Therefore, extended analysis of the targets identified in this study in human samples may reveal a novel function of Region 2 in HCC prognosis. Integration of different HBx mutants into the host genome was reported to be responsible for altered carcinogenesis in different patients [34]. We further suggest that patient-specific prognosis may also depend on this variable genomic integration. 

Previously, different parts of HBx protein were shown to behave differently to chemotherapy, indicating the possibility of designated roles for specific parts of HBx [35]. Based on this information, the putative HBV “Region 2” (amino acids 117 to 138) may have a functional impact on HCC development through genetic or epigenetic mechanisms. Moreover, “Region 2”, is an overlapping region for HBx (nt. 1374–1835), HBV core promoter (nt. 1613–1849) and Enhancer II (nt. 1627–1774), as well as a binding site for several transcription factors that regulate viral transcription, including HNF3, HNF4, TBP, Sp1 [36,37,38]. This region also seems to be a hotspot for high-risk HCC mutations [38] having clinical impact on chronic HBV and HCC [37]. Therefore, investigation of the regulatory roles of this region both in HBV and the human genome could have translational value in terms of differential diagnosis and prognosis of virally induced liver cancers. 

A high sequence homology of “Region 2” in different HBV genotypes with different intronic regions in the human genome is a serendipitous finding of this study. Although HBV integration is known to occur randomly throughout the entire human genome, it may also show some genotype-dependent pattern [39,40]. Besides well-defined preferred integration regions [41], viral insertion preference into variable locations in the host genome through this specific HBx region for different HBV genotypes is a novel observation. 

Based on protein homology analysis, specific parts of two proteins, Mpp7 and TGF β-LTBP, which affect EMT processes, were revealed to share high homology with Region 2. Mpp7 plays a role in the formation of tight junctions between epithelial and endothelial cells, which are crucial for cancer progression and metastasis [42,43,44], as well as in the apicobasal polarity in epithelial tissues. As the loss of cell-to-cell contacts is prerequisite for both EMT and epithelial cell polarity [45,46], altered apicobasal polarity is one of the initial modifications observed during carcinogenic transformation [47,48]. TGF-β has dual effects on HCC cancer cells by both suppressing or stimulating tumor development [12,49,50]. EMT and enhancement of cell migration and invasion, along with the differential effects of STIM1 on TGFβ-induced EMT, appear to be correlated with elevated Ca^2+^ influx via SOCE, as shown in breast cancer cells [51]. Along with an increased proliferation rate, as observed in our previous study [7], STIM1- and/or Orai1-overexpressed HCC CSCs appeared to be in an interim state favoring mesenchymal epithelial transition (MET). Significant elevation in epithelial marker E-cadherin along with upregulated mesenchymal markers (N-Cadherin and vimentin), presenting a partial EMT behavior, suggested that *STIM1* and *Orai1* enhancement leads CSCs into the second phase (equilibrium) of the 3Es (elimination, equilibrium and escape) of the cancer immunoediting process, facilitating a shift from EMT to MET, depending on the microenvironment [52]. Significantly upregulated SOCE-related cytosolic Ca^2+^ levels [7] appeared to be responsible for the enhanced invasion pattern of *STIM1*- and *Orai1*-enhanced CSCs, as *STIM1* enhancement, per se, inhibited invasion drastically.

Using a single cell line could lead to cell-specific patterns being identified rather than a general phenomenon. The Huh-7 HCC cell line in this study was selected among other HCC cell lines because it contains subpopulations of tumor-initiating stem cell-like cells [4,5,6], and is a cell line that can support viral replication when transfected with HBV DNA [53]. Therefore, the present data do not exclude the possibility that the HBV may modulate gene expression patterns differently in other liver cell lines. However, identifying such puzzle pieces, even from a single cell line, could help uncover complex molecular events in HCC. Further confirmation studies, including the silencing of STIM1 and Orai1, should be performed in order to assess the expression pattern of miR3653. Furthermore, performing these experiments in other cell lines that can support viral replication when transfected with HBV DNA, and evaluation of the expression of miR3653 in human tumor samples infected with HBV, are among the studies projected for the future. 

## 5. Conclusions

**Figure 6:**
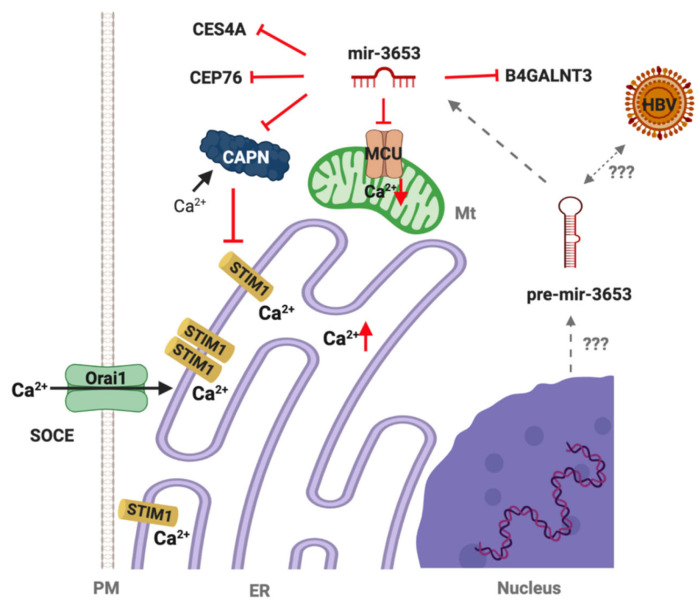
A schematic description of a proposed possible deception of a host defense system by miR3653 in *STIM1*- and/or *Orai1*-enhanced Huh-7 HCC CSCs. CAPN: calpain, a STIM-degrading Ca^2+^-dependent protease (downregulated); MCU: mitochondrial Ca^2+^ uniporter (downregulated); STIM1: ER Ca^2+^ sensor (upregulated); SOCE “store-operated Ca^2+^ entry” (upregulated by enhancement of both *STIM1* and *Orai1*). Ca^2+^ uptake into ER by SERCA (sarcoplasmic-endoplasmic Ca^2+^-ATPase) is not shown for simplicity. End result: cell survival, escape from apoptosis, suppressed invasion. (Created by BioRender.com).

In summary, we propose that miR3653 appears to be a part of the host defense mechanism recognized by HBV, and this mechanism may be mutually favorable for both the viral replication process and the host until the terminal stage of HCC. STIM1 upregulation may favor host cell survival by upregulating miR3653, which suppresses both calpain and MCU, further guaranteeing an uninterrupted cycle of protein machinery in the HBV-infected host cell (Figure 6). Furthermore, having this versatile genetic repertoire, HBx appears to be a “master key” in virulence as well as virus-driven epigenetic regulation of hepatocarcinogenesis, as HBV “Region 2” remnants were found in the human genome in a genotype-dependent manner. A possible “survival strategy” of HBV-induced liver CSCs might have been developed throughout the evolution, possibly via genome editing acquired throughout the long-lasting interaction with human cells. Together our data raises the question of whether each HBV genotype follows a different epigenetic strategy in carcinogenesis. Therefore, identification of the HBV genotype in infections may reveal how EMT is regulated in HCC progression and be useful for further therapeutic strategies.

## Data Availability

Data can be obtained by contacting the corresponding authors upon reasonable request.

## Supplementary Materials

The following supporting information can be downloaded at: https://www.mdpi.com/article/10.3390/jpm12122065/s1. Figure S1: A scheme for the workflow of study; Figure S2: Functional analyses on *STIM1*- and/or *Orai1*-enhanced Huh-7 HCC CSCs. (A) *E-Cadherin* mRNA expression after enhancement of *STIM1* and/or *Orai1*. *E-Cadherin* transcription levels were normalized to *18S rRNA* levels (target gene/18S rRNA mRNA × 10^2^, * *p* < 0.05, control vs. *STIM1*-OE; # *p* < 0.05, control vs. *STIM1*- & *Orai*1-OE; Student’s *t* test, unpaired, n = 4). Control: transfected with plasmid (PCMV6) only; OE: overexpression. (B) Invasion characteristics of *STIM1*-OE *or STIM1*&*Orai1*-OE Huh-7 HCC CSCs. ** *p* < 0.01, control vs. *STIM1*-OE; # *p* < 0.05, *STIM1*-OE vs. *STIM1*- & *Orai1*-OE; Student’s *t* test, unpaired, n = 8). Insert: Microscopic image of Huh-7 HCC CSCs run on invasion assay. Black arrows show cells (purple) that crossed 0.8-micrometer-diameter pores (red arrow) on matrigel membrane-containing well inserts. (Scale bar, 200 µm, marker 10× objective, Olympus IX71). HCC CSCs: hepatocellular carcinoma stem-like cells; OE: overexpressed; Figure S3: RNA Expression Values of different HCC cell lines in Liver Cancer Cell Line Database; Table S1: The list of miRNAs whose expressions were significantly changed upon *STIM1* and/or *Orai1* enhancement to mimic poor cancer prognosis in hepatocellular carcinoma (HCC). Table S2: HBV genotype dependent sequences of ‘Region 1’ and ‘Region 2’. Table S3: BLAT results of HBV Region 2 against human genome. HBV Region 2 of different genotypes has been found to be integrated in different intronic regions of different genes within human genome. Table S4: Oligonucleotide sequences of qRT PCR primers used to investigate EMT and MET properties of STIM1- and/or Orai1-enhanced Huh-7 HCC CSCs. HCC CSCs: hepatocellular carcinoma stem-like cells. Table S5: The list of GEO datasets of HCC lines analyzed for *MPP7* and *β-LTBP* expressions at the RNA level.
