# Supplementary material for: Implications of Possible HBV-Driven Regulation of Gene Expression in Stem Cell-like Subpopulation of Huh-7 Hepatocellular Carcinoma Cell Line
Source: J Pers Med. 2022 Dec 14;12(12):2065. doi: 10.3390/jpm12122065 (PMC9786676; doi:10.3390/jpm12122065)
Supplement: Supplementary file 1 [file jpm-12-02065-s001.zip › Supplementary Table S1.pdf]

Supplementary Table S1: The list of miRNAs of which their expressions were significantly changed upon STIM1 and/or Orai1 enhancement to mimic poor cancer prognosis in Hepatocellular carcinoma (HCC). OE: Overexpression.

Comparison 1: Stim1 OE Cancer Stem Cell (CSC) compared to Control (plasmid only) Cancer Stem Cell (CSC)

miRNA	Taget Mean	Target Std. Dev.	Control Mean	Control Std. Dev.	P value	Fold change
**mir-3653_(Mature_3')**	6.595154638	0.565466249	3.676764085	1.792694967	0.041692138	2.918390554
**mir-3651_(Mature_3')**	8.539157515	0.193430013	6.309877124	0.853272639	0.011473394	2.229280391
**mir-5001_(Mature_5')**	5.424543803	0.440915119	3.402232559	0.826013482	0.009223383	2.022311244
**mir-6715a_(Mature_3')**	6.474286217	0.519160512	4.66282746	0.949428813	0.022777997	1.811458758
**mir-3607_(Mature_3')**	10.43273726	0.26829328	8.653426108	0.825240993	0.018084146	1.779311156
**mir-576_(Mature_3')**	5.483775513	0.8994546	7.222120385	0.3759195	0.023285576	-1.738344873
**mir-101-1_(Mature_5')**	3.432632377	0.639184996	5.229857428	0.153554407	0.008993495	-1.797225052

Comparison 2: Orai1 OE Cancer Stem Cell (CSC) compared to Control (plasmid only) Cancer Stem Cell (CSC)

miRNA	Taget Mean	Target Std. Dev.	Control Mean	Control Std. Dev.	P value	Fold change
**mir-3653_(Mature_3')**	6.569538865	1.297740862	3.676764085	1.792694967	0.043534257	2.892774781
**mir-5001_(Mature_5')**	5.334951092	0.641612557	3.402232559	0.826013482	0.011280003	1.932718533
**mir-1538_(Mature_3')**	6.512744271	0.797275217	4.779650704	0.563818	0.014406629	1.733093567
**mir-6798_(Mature_3')**	6.753374818	0.468498981	5.084659615	0.193220785	0.00277273	1.668715203
**mir-8485_(Mature_3')**	7.435736449	0.552563101	5.793276294	0.23640102	0.005209497	1.642460155
**mir-197_(Mature_5')**	7.864427359	0.485503679	6.264176248	0.209466464	0.003527529	1.600251111
**mir-6715a_(Mature_3')**	6.261465064	0.735855607	4.66282746	0.949428813	0.039693683	1.598637604

**mir-4479_(Mature_3')**	4.986406538	0.889461272	3.423393283	0.721377666	0.035686821	1.563013255
**mir-4512_(Mature_3')**	6.663321218	0.203372369	5.135294838	0.283432362	0.000207446	1.52802638
**mir-548at_(Mature_5')**	3.350986325	0.665914135	4.869249079	0.676766358	0.018649172	-1.518262754
**mir-548b_(Mature_5')**	5.945021252	0.505601042	7.527855889	0.459495246	0.003648395	-1.582834637
**mir-548d-1// mir-548d-2_(Mature_5')**	3.586872274	1.011594701	5.173795881	0.4118418	0.044288945	-1.586923607
**mir-451a_(Mature_5')**	4.78893661	0.482888588	6.486764737	0.412088144	0.00188577	-1.697828126
**mir-26a-1_(Mature_3')**	5.616966521	0.462203057	7.762631138	0.311636756	0.000468479	-2.145664617

Comparison 3: EpCAM & CD133 Positive Cancer Stem Cell (CSC) compared to EpCAM & CD133 negative Non-cancer Stem Cells (NCSC)

miRNA	Taget Mean	Target Std. Dev.	Control Mean	Control Std. Dev.	P value	Fold change
**mir-552_(Mature_3')**	9.10144	0.44581	7.25759	0.21636	0.00126	1.84385
**mir-6735_(Mature_5')**	4.50756	0.81081	2.75537	0.75831	0.01977	1.75219
**mir-552_(Mature_5')**	7.05840	0.44170	5.39928	0.33686	0.00126	1.65912
**mir-3194_(Mature_5')**	6.26785	0.81847	4.70208	0.57609	0.02351	1.56577
**mir-4461_(Mature_3')**	2.73261	0.44740	4.23348	0.55608	0.00624	-1.50087
**mir-3150a_(Mature_5')**	3.85441	0.92168	5.44836	0.29278	0.03511	-1.59394
**mir-365a_(Mature_5')**	4.92110	0.38865	6.56542	0.77851	0.01628	-1.64432
**mir-125b-1_(Mature_3')**	7.34214	0.25193	9.19897	0.34637	0.00021	-1.85683
**mir-181d_(Mature_5')**	6.54752	0.26773	8.45216	0.16055	0.00007	-1.90463
**mir-3591_(Mature_3')**	7.14796	0.48494	9.05504	0.19899	0.00193	-1.90708
**mir-181c_(Mature_5')**	6.29427	0.30647	8.47269	0.19584	0.00006	-2.17842

**mir-1261_(Mature_5')**	4.13906	0.85072	6.53759	0.06144	0.01081	-2.39853
