# Supplementary material for: Implications of Possible HBV-Driven Regulation of Gene Expression in Stem Cell-like Subpopulation of Huh-7 Hepatocellular Carcinoma Cell Line
Source: J Pers Med. 2022 Dec 14;12(12):2065. doi: 10.3390/jpm12122065 (PMC9786676; doi:10.3390/jpm12122065)
Supplement: Supplementary file 1 [file jpm-12-02065-s001.zip › Supplementary Table S2.pdf]

Supplementary Table S2: HBV genotype dependent sequences of ‘Region 1’ and ‘Region 2’.

**Hepatitis B Virus Genotype**	**Sequence of the viral genome regions where stemloop sequences of miRNAs are mostly aligned aligned**	
	**Region 1**	**Region 2**
**A**	GATCCTTCGCGGGACGTCCTTTGTTTACGTCCCGTCGGCGCTGAATCCCGCGGACGACCCCTCTCGGGGCC	TGTTTAAGGACTGGGAG_GAGCTGGGGGAGGAGATTAGGTTAATGATCTTTGTATTAGTAAATTG
**B**	GATCCTGCGCGGGACGTCCTTTGTCTACGTCCCGTCGGCGCTGAATCCCGCGGACGACCCCTCCCGGGGCC	TGTTTACTGAGTGGGAG_GAGTTGGGGGAGGAGATCAGGTTAAAGGTCTTTGTACTAGTAAATTG
**C**	GATCCTGCGCGGGACGTCCTTTGTCTACGTCCCGTCGGCGCTGAATCCCGCGGACGACCCGTCTCGGGGCC	TGTTTAAGGACTGGGAG_GAGTTAGGGGAGGAGACTAGGTTAATGATCTTTGTACTAGTAAATTG
**D**	GATCCTGCGCGGGACGTCCTTTGTTTACGTCCCGTCGGCGCTGAATCCCGCGGACGACCCTTCTCGGGGCC	TGTTTAAGGACTGGGAG_GAGTTGGGGGAGGAGACTAGATTAATGATCTTTGTACTAGTAAATTG
**E**	GATCCTGCGAGGGACGTCCTTTGTCTACGTCCCGTCAGCGCTGAATCCTGCGGACGATCCGTCTCGGGGTC	TGTTTAAAGACTGGGAG_GAGTTGGGGGAGGAGATTAGATTAAAGATCTTTGTACTAGTAAATTG
**F**	GATCCTGCGCGGGACGTCCTTTGTTTACGTCCCGTCGGCGCTGAATCCCGCGGACGACCCTTCCCGGGGTC	TATTTAAGGACTGGGAG_GAGCTGGGGGAGGAGATCAGGTTAAAGGTCTTTGTACTAGTAAATTG
**G**	GATCCTTCGCGGGACGTCCTTTGTTTACGTCCCGTCAGCGCTGAATCCAGCGGACGACCCCTCCCGGGGCC	TTTTTGCTGAGTGGGAA_GAATTAGGCAATGAGTCCAGGTTAATGACCTTTGTATTAGTAAATTG
**H**	GATCCTGCGCGGGACGTCCTTTGTCTACGTCCCGTCGGCGCTGAATCCTGCGGACGACCCCTCTCGTGGTC	TATTTAAGGACTGGGAG_GAGTCGGGGGAGGAGTTGAGGTTAAAGGTCTTTGTACTAGTAAATTG
