# Supplementary material for: Implications of Possible HBV-Driven Regulation of Gene Expression in Stem Cell-like Subpopulation of Huh-7 Hepatocellular Carcinoma Cell Line
Source: J Pers Med. 2022 Dec 14;12(12):2065. doi: 10.3390/jpm12122065 (PMC9786676; doi:10.3390/jpm12122065)
Supplement: Supplementary file 1 [file jpm-12-02065-s001.zip › Supplementary Table S3.pdf]

Supplementary Table S3 BLAT results of HBV Region 2 against human genome. HBV Region 2 of different genotypes has been found to be integrated in different intronic regions of different genes within human genome.

	**Viral Sequence Putative "Region 2"**				**Human Chromosomal Region Information**					
**Hepatitis B Virus Genotype**	**BLAT Score**	**Start Position**	**End Position**	**Sequence Identitiy Percentage**	**Chromosome number**	**Strand**	**Start position**	**End Position**	**Genes within the corresponding chromosomal region**	**Location within the gene where the viral genomic region found**
**A**	22	8	30	100%	16	-	66992169	66992192	*CES4A*	Intron (1/11)
	21	11	33	95.7%	1	-	46522501	46522523	Alternate haplotype sequence	
	20	13	32	100%	8	-	127426802	127426821	*CASC8*	Intron (4/5)
**B**	23	7	30	100%	2	-	30732664	30732688	*CAPN13*	Intron (19/22)
	21	9	31	95.7%	18	-	5564745	5564767	*EPB41L3*	Intron (3/21)
	20	15	34	100%	20	-	38727067	38727086	*SLC32A1*	Intron (1/1)
	20	11	30	100%	18	-	12668263	12668282	*PSMG2*<br>*CEP76*	Intron (1/6)<br>Intron (12/12)
	23	13	38	96%	2	+	240731950	240731976	*KIF1A*<br>*Alt haplotypes*	Intron (36/46)<br>Intron (38/48)<br>Intron (36/47)<br>Intron (37/47)<br>Intron (38/49)
	20	13	32	100%	1	-	18575914	18575933	Alternate haplotype sequence	
	20	11	32	95.5%	3_KI270937v1_alt	+	81170	81191	*MUC20-OT1*	Intron (2/4)<br>Intron (2/3)

**C**										Intron(1/2)<br>Intron (3/4)
	20	11	32	95.5%	3_KI270936v1_alt	+	80903	80924	*MUC20-OT1*	Intron (2/4)<br>Intron (2/3)<br>Intron(1/2)<br>Intron (3/4)
	20	11	32	95.5%	3_KI270934v1_alt	+	80596	80617	*MUC20-OT1*	Intron (2/4)<br>Intron (2/3)<br>Intron(1/2)<br>Intron (3/4)
**D**	21	7	27	100%	17	-	38850927	38850947	*RPL23*<br>*Alt haplotypes*	Intron (3/4)<br>Intron (3/5)<br>Intron (3/3)
	21	15	35	100%	12	+	2737703	2737723	Alternate haplotype sequence	
	20	16	35	100%	12	+	505803	505822	*B4GALNT3*	Intron (1/19)
**E**	23	25	48	100%	3_KI270778v1_alt	-	233459	233497	Alternate haplotype sequence	
	20	15	34	100%	20	-	38727067	38727086	*SLC32A1*	Intron (1/1)
**F**	22	8	30	100%	1	-	66992169	66992192	*CES4A*	Intron (1/11)
	21	11	33	95.7%	1	-	46522501	46522523	Alternate haplotype sequence	
	20	13	32	100%	8	-	127426802	127426821	*CASC8*	Intron (4/5)
