# Supplementary material for: Implications of Possible HBV-Driven Regulation of Gene Expression in Stem Cell-like Subpopulation of Huh-7 Hepatocellular Carcinoma Cell Line
Source: J Pers Med. 2022 Dec 14;12(12):2065. doi: 10.3390/jpm12122065 (PMC9786676; doi:10.3390/jpm12122065)
Supplement: Supplementary file 1 [file jpm-12-02065-s001.zip › Supplementary Table S4.pdf]

Supplementary Table S4 Oligonucleotide sequences of RT PCR primers used to investigate EMT and MET properties of STIM1- and/or Orai1-enhanced Huh-7 HCC CSCs.

**Gene**		**Primer sequence (5'-3')**	**Amplicon size (bp)**
**E-Cadherin**	F	TAC ACT GCC CAG GAG CCA GA	103
	R	TGG CAC CAG TGT CCG GAT TA	
**N-Cadherin**	F	ATT GGA CCA TCA CTC GGC TTA	159
	R	CAC ACT GGC AAA CCT TCA CG	
**Vimentin**	F	CCT TGA CAT TGA GAT TGC CAC CTA	215
	R	TCA TCG TGA TGC TGA GAA GTT TCG	
**18S rRNA**	F	CGA CGA CCC ATT CGA ACG TCT	312
	R	GCT ATT GGA GCT GGA ATT ACC G	
