# Supplementary material for: Implications of Possible HBV-Driven Regulation of Gene Expression in Stem Cell-like Subpopulation of Huh-7 Hepatocellular Carcinoma Cell Line
Source: J Pers Med. 2022 Dec 14;12(12):2065. doi: 10.3390/jpm12122065 (PMC9786676; doi:10.3390/jpm12122065)
Supplement: Supplementary file 1 [file jpm-12-02065-s001.zip › Supplementary Table S5.pdf]

Table S5. The list of GEO Datasets of Hepatocellular Carcinoma Cell Lines analyzed for Mpp7 and β-LTBP expressions at the RNA level.

GEO Accession Number	Probe ID for Mpp7	Probe ID for β-LTBP (LTBP 2)
GSE112788	1564308_a_at	202728_s_at
GSE79232 (GPL16700 Platform)	MPP7	No data
GSE97172 (GPL6244 Platform)	7932765	7980152
GSE85274 (GPL13667 Platform)	11734214_a_at	11720527_at
GSE36139 (GPL15308 Platform)	143098_at	4053_at
