# Supplementary figures and images for: Implications of Possible HBV-Driven Regulation of Gene Expression in Stem Cell-like Subpopulation of Huh-7 Hepatocellular Carcinoma Cell Line
Source: J Pers Med. 2022 Dec 14;12(12):2065. doi: 10.3390/jpm12122065 (PMC9786676; doi:10.3390/jpm12122065)

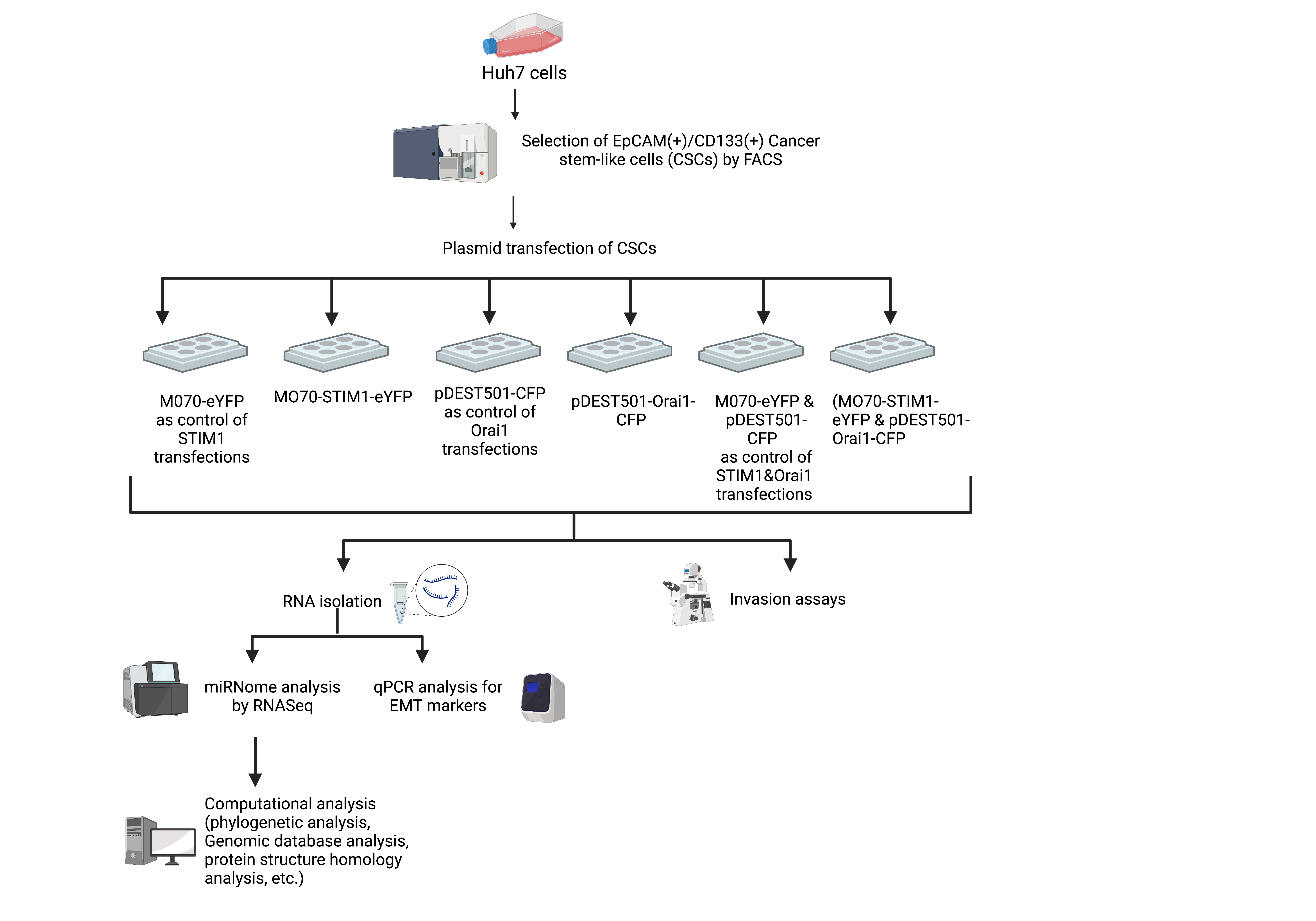

Supplement: Supplementary file 1 [file jpm-12-02065-s001.zip › Supplementary Figure S1.png]

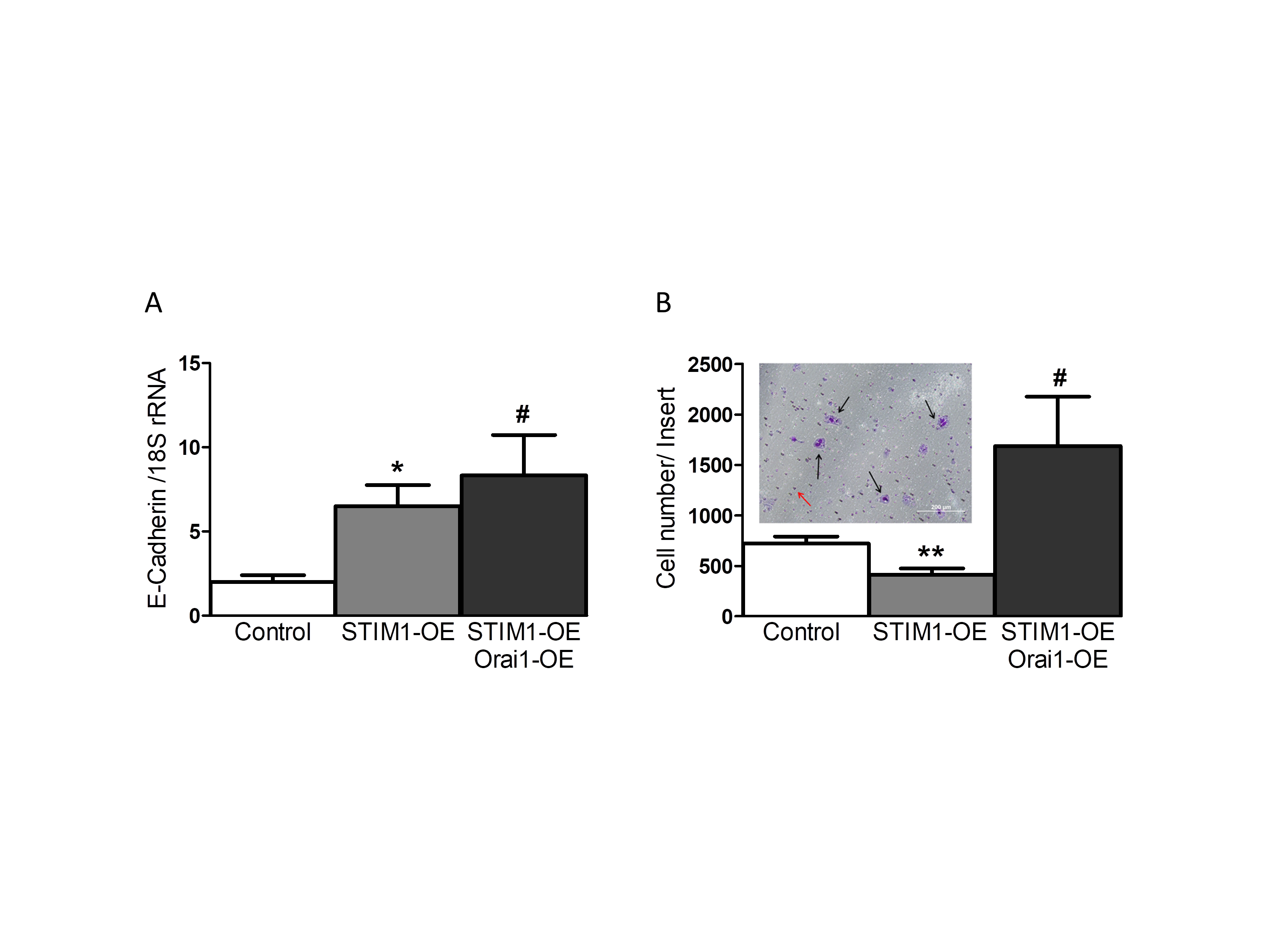

Supplement: Supplementary file 1 [file jpm-12-02065-s001.zip › Supplementary Figure S2.tif]

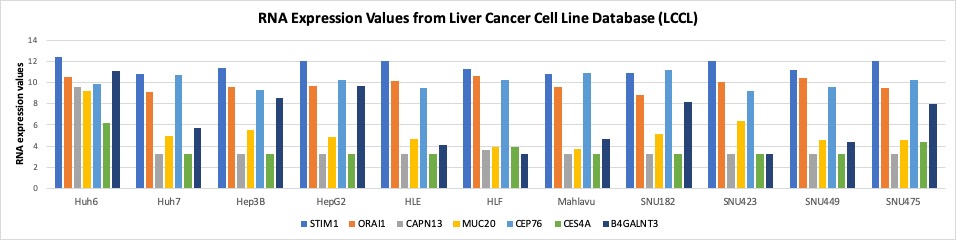

Supplement: Supplementary file 1 [file jpm-12-02065-s001.zip › Supplementary Figure S3.tif]
